# Graded extent of hippocampal resection is related to neuropsychological outcomes in temporal lobe epilepsy surgery

**DOI:** 10.1002/epi.70162

**Published:** 2026-02-26

**Authors:** Eliza M. Reedy, Emma Robinson, Thandar Aung, Catherine Liégeois‐Chauvel, Danielle R. Carns, Natalie Sherry, Luke C. Henry, Bradford Z. Mahon, Arka N. Mallela, Jorge A. Gonzalez‐Martinez

**Affiliations:** ^1^ Department of Neurological Surgery University of Pittsburgh/University of Pittsburgh Medical Center Pittsburgh Pennsylvania USA; ^2^ Department of Neurological Surgery Cornell University Ithaca New York USA; ^3^ Department of Neurology University of Pittsburgh/University of Pittsburgh Medical Center Pittsburgh Pennsylvania USA; ^4^ Department of Psychology Carnegie Mellon University Pittsburgh Pennsylvania USA; ^5^ Neuroscience Institute Carnegie Mellon University Pittsburgh Pennsylvania USA; ^6^ Department of Neurological Surgery Rush University Medical Center Chicago Illinois USA

**Keywords:** anterior temporal lobectomy, hippocampus, memory, neuropsychology, surgery

## Abstract

**Objective:**

Surgical resection for epilepsy seeks to maximize seizure freedom while minimizing new neurocognitive impairments. Tailored resections guided by anatomoelectroclinical (AEC) hypotheses offer the possibility of sparing parts of the hippocampus. The relationship between the extent of hippocampal resection and postoperative neurocognitive outcomes in this context has not been studied and has important implications for clinical practice. We test this relationship in a series of left and right tailored anterior temporal lobectomy (ATL) surgeries.

**Methods:**

We conducted a retrospective analysis of 34 adult patients with drug‐resistant temporal lobe epilepsy (18 left, 16 right) who underwent tailored ATL based on individualized AEC hypotheses at the University of Pittsburgh Medical Center. All patients completed standardized pre‐ and postoperative neuropsychological testing, and 85.3% underwent preoperative stereoelectroencephalography to guide resection. Surgical extent was tailored through a multidisciplinary process integrating AEC correlations and intraoperative electrophysiology. Preoperative and postoperative hippocampal volumes were measured and correlated with changes in verbal and visual memory, as well as language performance.

**Results:**

Greater extent of resection of the left hippocampus was significantly associated with worse postoperative outcomes in both verbal and visual recall. Extent of resection of the right hippocampus was not related to reductions in performance across any domain, with some indication of improvements in performance after right ATL surgery at the group level. Seizure outcomes (66.6% Engel I at 2 years) were consistent with the existing literature and did not vary with hippocampal resection extent.

**Significance:**

These findings highlight the critical role of the left hippocampus in supporting both verbal and visual memory and underscore the importance of preserving hippocampal tissue during left ATL when feasible. Our results support the utility of AEC‐guided tailored resections as a strategy to balance seizure control with cognitive preservation.

**Key points**

- Extent of AEC‐guided left hippocampal resection is correlated with changes in cognitive performance after tailored ATL.
- Sparing nonepileptogenic hippocampal tissue is associated with a relative cognitive benefit.
- Tailored hippocampal resections protect cognitive function while preserving seizure relief.

## 1 INTRODUCTION

Surgical treatment for epilepsy, particularly anterior temporal lobectomy (ATL), remains a cornerstone therapy for patients with drug‐resistant temporal lobe epilepsy, offering seizure freedom rates between 62% and 75%.^1^, ^2^, ^3^ Such surgical treatments frequently involve resection of the hippocampus, a procedure that has long illuminated the role of the hippocampus in cognition. A canonical example is that of patient H.M., whose bilateral mesial temporal resection resulted in a profound anterograde amnesia, informing decades of neuropsychological research in memory.^4^ It is now well established that seizure freedom and cognitive outcomes interact to influence postoperative quality of life.^2^, ^5^ As a result, understanding the cognitive functions of the hippocampus remains critical to epilepsy surgery and its goal of balancing good seizure outcomes with good cognitive outcomes.

Many foundational investigations of the cognitive impact of hippocampal resection have been based on an all‐or‐nothing surgical approach, leaving uncertainty regarding the impact of partial hippocampal resections. For instance, one common approach has been to compare the cognitive outcomes of patients undergoing ATL with hippocampal resection versus ATL resection without hippocampal resection.^6^, ^7^ A criticism of this comparison method is that ATL with hippocampal resection damages white matter and other structures in the temporal stem, confounding interpretation of the contribution of the hippocampus itself.^8^ Recent studies have instead compared traditional open ATL surgery with more targeted or less invasive techniques such as selective amygdalohippocampectomy (SAH) and stereotactic laser amygdalohippocampotomy.^9^, ^10^ Although these techniques cause less collateral damage to temporal stem structures, they reflect the same binary perspective; either the hippocampus is resected/ablated, or it is left intact.

Considering extent of hippocampal resection as a continuous variable is critical in the context of changing surgical practices. Contemporary surgical approaches increasingly emphasize precision and individualized, network‐informed resections, supported by advances in invasive electrophysiological monitoring.^11^ Stereoelectroencephalography (SEEG) provides three‐dimensional mapping of seizure onset zones, allowing differentiation between epileptogenic hippocampal subregions such as head, body, and tail.^12^, ^13^ Some studies have already called for preservation of the hippocampal tail or body when possible, particularly for patients with a radiologically normal hippocampus.^14^, ^15^ Furthermore, research using functional magnetic resonance imaging (fMRI) has found that the posterior remnants of resected hippocampi contribute to memory preservation following ATL.^16^

Two key empirical questions remain to be answered in the context of SEEG‐guided, individually tailored hippocampal resection. First, is there a dose‐dependent relationship between extent of hippocampal resection and cognitive outcome after surgery? Second, can seizure freedom outcomes be maintained at the clinical standard even when some hippocampal tissue is preserved?

Answering these questions has several implications. The first is the clinical consideration of whether it is justified to preserve a partial hippocampus. If neuropsychological outcomes are dependent on the amount of hippocampus removed, without a change in seizure relief from the surgery, this would be a strong justification for individually tailoring the extent of hippocampal resection. The second is the basic scientific consideration of what cognitive functions are truly related to the hippocampus per se, as opposed to nearby structures, such as temporal stem white matter, which are damaged in accessing the hippocampus. Third and finally, studying hippocampal resection as a continuous variable enables better functional dissection of hippocampal subregions. It is well recognized that anterior and posterior portions of the hippocampus support dissociable aspects of memory encoding and retrieval.^17^, ^18^, ^19^ Resection of such a functionally heterogenous structure should be considered as more than a binary phenomenon.

Here, we address this gap via a single‐center cohort study, examining neuropsychological outcomes in patients undergoing left or right ATL with SEEG‐guided tailored hippocampal resections. We quantify the relationship between postoperative memory outcomes and the percentage of hippocampal tissue resected, testing two hypotheses: (1) that smaller hippocampal resections are associated with better memory outcomes and (2) that smaller hippocampal resections are not associated with worse seizure outcomes. We demonstrate that extent of hippocampal resection is related to changes in verbal and visual memory performance after surgery, with smaller resections resulting in less impaired memory. We also observe that seizure outcomes are comparable to the published clinical standard. Our findings inform precision epilepsy surgery strategies that balance optimal seizure control with preservation of cognitive function.

## 2 MATERIALS AND METHODS

### 2.1 Participants

This study was approved by the University of Pittsburgh's institutional review board (IRB; STUDY21020058). Data were prospectively compiled and managed using REDCap as part of a standard data protocol.^20^ For this retrospective analysis, patients were selected from the REDCap database according to the following criteria: (1) 18 years or older at the time of their temporal lobe resection, (2) English‐speaking, (3) no prior resection or hippocampal ablation, and (4) completed both preoperative and postoperative neuropsychological testing at University of Pittsburgh Medical Center (UPMC).

Thirty‐four drug‐resistant temporal lobe epilepsy patients who underwent anterior temporal lobe resection (18 left temporal lobe, 16 right temporal lobe) at UPMC were enrolled with IRB approval from the University of Pittsburgh. All cases were discussed in the multidisciplinary epilepsy patient management conference (MEPMC) before invasive monitoring or resection. Twenty‐nine of 34 (85.3%) patients underwent SEEG exploration prior to their resection, according to the decision of the MEPMC. Nineteen of 34 (55.9%) underwent magnetoencephalographic (MEG) evaluation of verbal memory function. Of these 19, all were determined to have verbal memory dominance in the left hemisphere. Group consensus of the MEPMC deemed all 34 patients to have language and verbal memory presence in the left hemisphere based on semiology and neuropsychological testing.

Decision to undergo resection was based on group consensus of the MEPMC in accordance with each patient's consent after extensive counseling on possible seizure and cognitive outcomes from surgery. Anatomoelectroclinical (AEC) hypotheses were generated for each patient following the process outlined by Mallela et al.^21^ All patients included in this study had at least one such hypothesis that included the mesial temporal lobe; no bilateral patients were included in this analysis.

Patient demographic and clinical data were obtained from the electronic medical record. Preoperative MRIs were examined and classified as lesional versus nonlesional by a board‐certified neuroradiologist as well as the group consensus of the MEPMC. Seizure freedom outcomes were recorded and classified using the Engel classification 2+ years postoperatively for 27 patients and at last follow‐up for the remaining eight, who did not have 2‐year follow‐up. All recorded seizure outcomes were at 6 months or more postsurgery.

### 2.2 Surgical approach and technique

Patients underwent a tailored temporal lobectomy based on the epileptogenic zone (EZ) as localized by clinicians (T.A., A.N.M., J.A.G.‐M.) and as has been previously described by this group.^21^ We describe the methods in brief here, and further detail can be found in this prior publication. The EZ was determined and extent of resection was planned using AEC correlation of semiology, neuroimaging, phase 1 electroencephalographic evaluation, neuropsychological testing, ancillary testing including MEG, interictal positron emission tomography, and ictal/interictal single photon emission computed tomography, and, if applicable, SEEG evaluation. SEEG implantation was determined based on group consensus and followed an orthogonal approach. The number and location of hippocampal SEEG electrodes for each patient can be found in Table S1. Both ictal and interictal SEEG data contributed; ictal onset defined the epileptogenic core, whereas interictal high‐frequency discharges and absence of normal hippocampus background activity delineated further epileptogenic areas to guide the posterior margin.

When ictal onsets and early propagation were confined to the anterior hippocampus and amygdala, the resection plan was limited to the anterior portions of the hippocampus (typically 2.5–3.0 cm from the temporal pole). In contrast, if ictal discharges or frequent interictal spikes extended into the posterior hippocampus, the resection plan was extended posteriorly (up to 4.0–4.5 cm). Intraoperatively, electrocorticographic (ECoG) recordings were performed in a stepwise manner, both before and after resection, to assess residual epileptiform activity. This provided confirmatory data and, in some cases, assisted in deciding between similar competing AEC hypotheses (e.g., hippocampal head vs. head and part of body), but ECoG did not prompt major divergence from the AEC hypothesis or set of hypotheses developed preoperatively by the clinical team.

**FIGURE 1:**
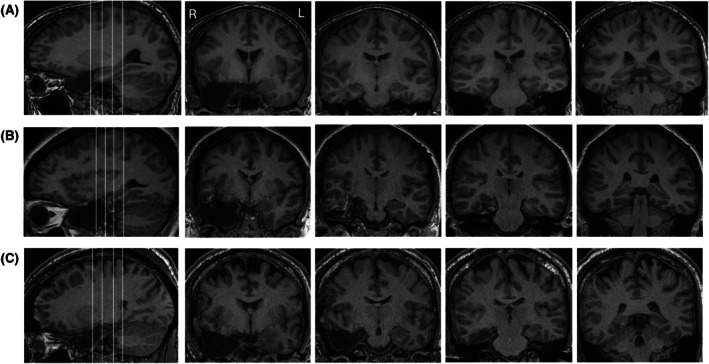
Representative examples of partial or complete hippocampal sparing. (A) Fully preserved hippocampus with resected amygdala. (B) Resection of the hippocampal head with preservation of the body and tail. (C) Resection of the hippocampal head and body with preservation of tail. L, left; R, right.

All patients underwent frontotemporal craniotomy with exposure of the temporal lobe. The extent of resection of the hippocampus in the anterior to posterior direction was based on the preoperative hypothesis developed by clinicians and guided by ongoing active review and discussion of intraoperative ECoG by the epileptologist and surgical team as described above. The extent of neocortical resection and the extent of lateral temporal resection were individually tailored and guided by intraoperative electrophysiology following the same stepwise process. Common aspects for all patients in this study are that the temporal pole and amygdala were resected, and that resection of mesial structures was confined laterally by the collateral sulcus. For representative examples of various extents of hippocampal resection, see Figure 1.

### 2.3 Neuropsychological measures

Neuropsychological evaluations were performed at a median of 5 months before and 7 months after resection. Verbal learning and memory were assessed using the Rey Auditory Verbal Learning Test,^22^ and visuospatial learning and memory were assessed using the Rey Complex Figure Test.^23^ Naming was assessed with the Boston Naming Test.^24^ Verbal fluency was assessed with the Delis–Kaplan Executive Function System Letter Fluency and Category Fluency subtests.^25^ Estimated premorbid functioning was assessed with the Wechsler Test of Adult Reading and the Vocabulary subtest of the Wechsler Adult Intelligence Scale. Vocabulary was only administered preoperatively, but all other tests were part of both preoperative and postoperative neuropsychological batteries. All neuropsychological scores were converted to age‐ and education‐matched *Z*‐scores according to published norms. The primary outcome of interest for each test was the change in *Z*‐score (postoperative minus preoperative) for each patient. A limitation of this measure is that changes in *Z*‐score for patients who were already at the extreme ends of the distribution do not necessarily represent a change in functional impairment. For this reason, pre‐ and postoperative *Z*‐scores were truncated at −4 and +4 so that clinically relevant changes are represented in our results.

### 2.4 Image acquisition and hippocampus segmentations

High‐resolution T1‐weighted anatomical images were acquired using a T1‐weighted magnetization‐prepared rapid acquisition gradient echo sequence on a Prisma 3‐T scanner (Siemens Healthineers). The imaging parameters were as follows: repetition time (TR) = 2000 ms, echo time (TE) = 3.17 ms, inversion time (TI) = 900 ms, flip angle = 8°, and field of view (FOV) = 256 × 256 mm^3^, with an isotropic voxel size of 1 × 1 × 1 mm^3^. In cases where the T1 image was unavailable or motion‐degraded, the high‐resolution fluid‐attenuated inversion recovery image was used instead. The sequence parameters were as follows: TR = 5000 ms, TE = 386 ms, TI = 1600 ms, and FOV = 256 × 256 mm^3^, with an isotropic voxel size of .9 × .9 × .9 mm^3^. Preoperative MRIs were acquired at a median of 6 months before surgery, and postoperative MRIs were acquired within 48 h following surgery. From these images, manual segmentation of the hippocampal volume was performed in ITK‐SNAP.^26^ Manual segmentations were used because automated segmentation methods were found to be inaccurate for postoperative MRIs due to the frequent presence of debris in the surgical field.

**FIGURE 2:**
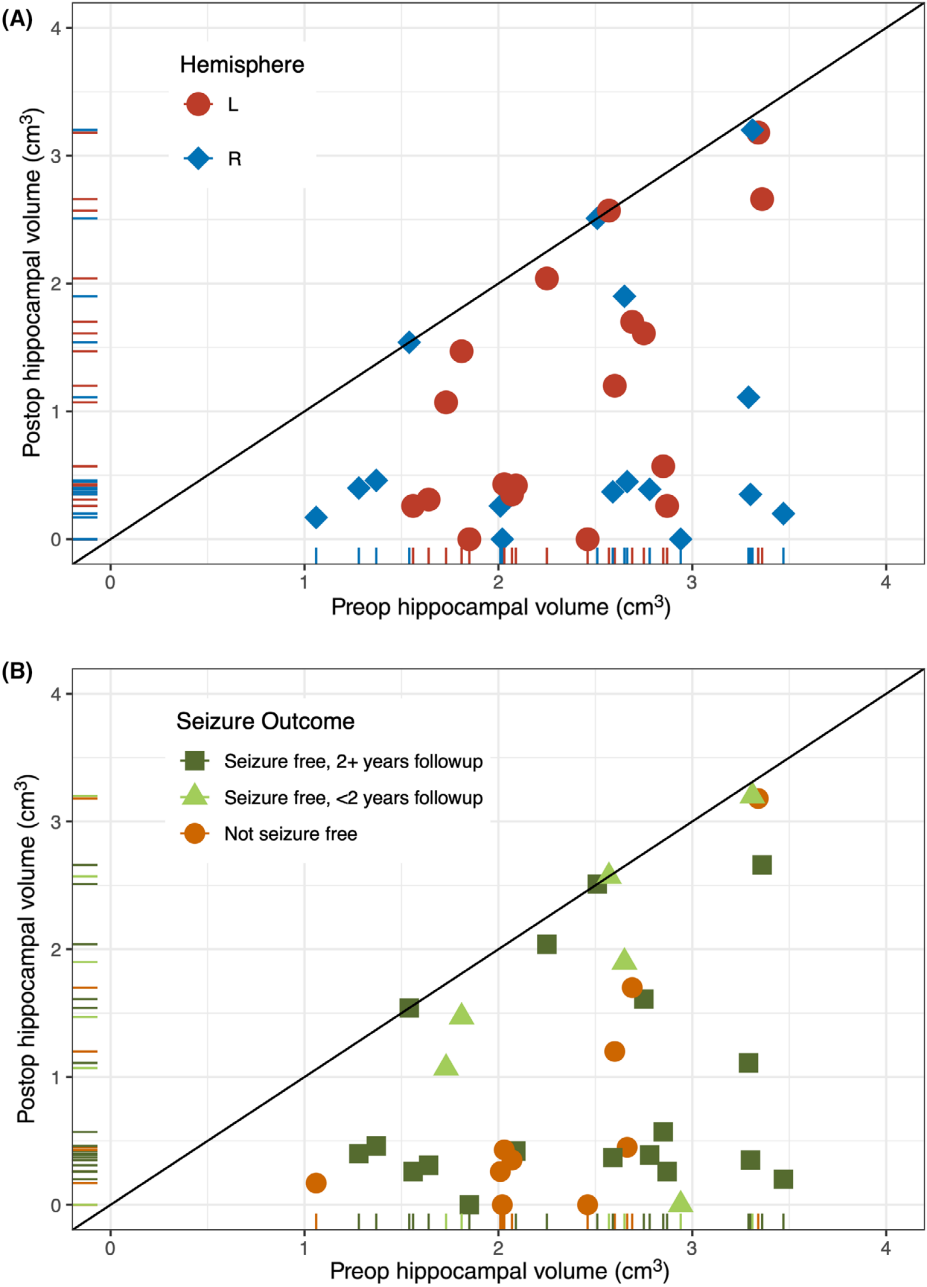
Preoperative and postoperative absolute hippocampal volumes for all participants. (A) Preoperative and postoperative hippocampal volumes colored by side of surgery. Preoperative and postoperative hippocampal volumes do not differ between right (R) ATL and left (L) anterior temporal lobectomy patients. (B) Preoperative and postoperative hippocampal volumes and seizure freedom. Hippocampal resection extent is not related to seizure outcome at a population level.

Segmentations were all performed by a rater who was blinded to neuropsychological performance. Segmentations were bounded anteriorly by the amygdala or surgical cavity and posteriorly by the fornix and did not include the fimbria of the fornix. These initial segmentations were then independently reviewed by a neurosurgeon (A.N.M., J.A.G.‐M.) who was also blinded to neuropsychological performance at the time. In the case of disagreement, the disagreement was discussed, and the rater performed a new segmentation, which was reviewed anew. This process was repeated as necessary until all parties agreed. In some cases where no hippocampus was resected, decompression of the tissue due to resection of surrounding structures led to a slight increase in volume of hippocampal tissue. In these cases, postoperative hippocampal volume was set to preoperative hippocampal volume, indicating that no hippocampal tissue was resected. For a scatterplot of all preoperative and postoperative absolute volumes, see Figure 2A. For representative examples of segmentations, see Figure 1B.

### 2.5 Statistical analysis

Continuous variables are reported as mean ± SD unless otherwise indicated, whereas categorical variables are reported as *n* (% of total). Categorical variables are reported as absolute counts and corresponding percentages relative to the total sample size. Linear regression was performed between neuropsychological variables of interest and percentage of hippocampal resection. Paired *t*‐tests were used as appropriate to compare preoperative and postoperative neuropsychological scores, and unpaired *t*‐tests were used for cross‐subject comparisons. For analyses involving multiple comparisons, *p*‐values were adjusted using false discovery rate (FDR) correction. An FDR‐corrected alpha of *q* < .05 was the prespecified as the threshold for statistical significance. Additionally, we report results that achieved nominal statistical significance at the uncorrected *p* < .05 level but did not survive FDR correction, to provide a complete view of potentially relevant trends. Statistical comparisons were performed in R.^27^ Deidentified data will be available with a DOI on Figshare at the time of publication, and imaging files are available by contacting the corresponding author.

## 3 RESULTS

### 3.1 Patient demographics, clinical variables, and hippocampal volumes

Patients had an average age of 36.8 ± 13.3 years. Their average age at seizure onset was 24.5 ± 17.0, with 12.3 ± 10 years of seizures prior to their resection. Average preoperative hippocampal volume was 2.39 ± .66 cm^3^, and average postoperative hippocampal volume was .98 ± .97 cm^3^. Individual pre‐ and postoperative absolute hippocampal volumes are displayed in Figure 2. Left ATL and right ATL patients did not significantly differ in preoperative hippocampal volume, postoperative hippocampal volume, or percent of hippocampal tissue resected (Table 1).

**TABLE 1:** Left ATL and right ATL patients did not differ in age at surgery, age at seizure onset, years of seizures, preoperative HV, postoperative HV, percent resected, HS, or seizure freedom rates.

	Left, *n* = 18		Right, *n* = 16		*T*	*p*
	Mean	SD	Mean	SD		
Age at surgery, years	40.6	14.6	32.7	10.7	1.81	.079
Age at seizure onset, years	29.1	18.0	19.5	14.8	1.71	.098
Years of seizures	11.5	9.29	13.3	11.1	−.50	.63
Preoperative HV, cm^3^	2.36	.55	2.42	.79	−.28	.80
Postoperative HV, cm^3^	1.11	.99	.83	.96	.85	.40
Percent resected	55.6	34.6	65.2	36.1	−.78	.44
	*n*	%	*n*	%	*χ* ^2^ (*df* = 1)	*p*
HS	4	22%	5	31%	.042	.84
Seizure freedom rate	12	66.6	12	75	.024	.88

Abbreviations: HS, hippocampal sclerosis; HV, hippocampal volume.

### 3.2 Preoperative hippocampal volumes and neuropsychological status

In left ATL, larger preoperative hippocampal volume was associated with better picture naming (*ß* = 1.4617, 95% CI = .50–2.42, adjusted *R* ^2^ = .37, *p* = .0053) and better vocabulary (*ß* = 1.31, 95% CI = .48–2.189, adjusted *R* ^2^ = .38, *p* = .0058). Preoperative hippocampal volume was not associated with any of the studied neuropsychological scores in right ATL.

### 3.3 Neuropsychological change after surgery

**FIGURE 3:**
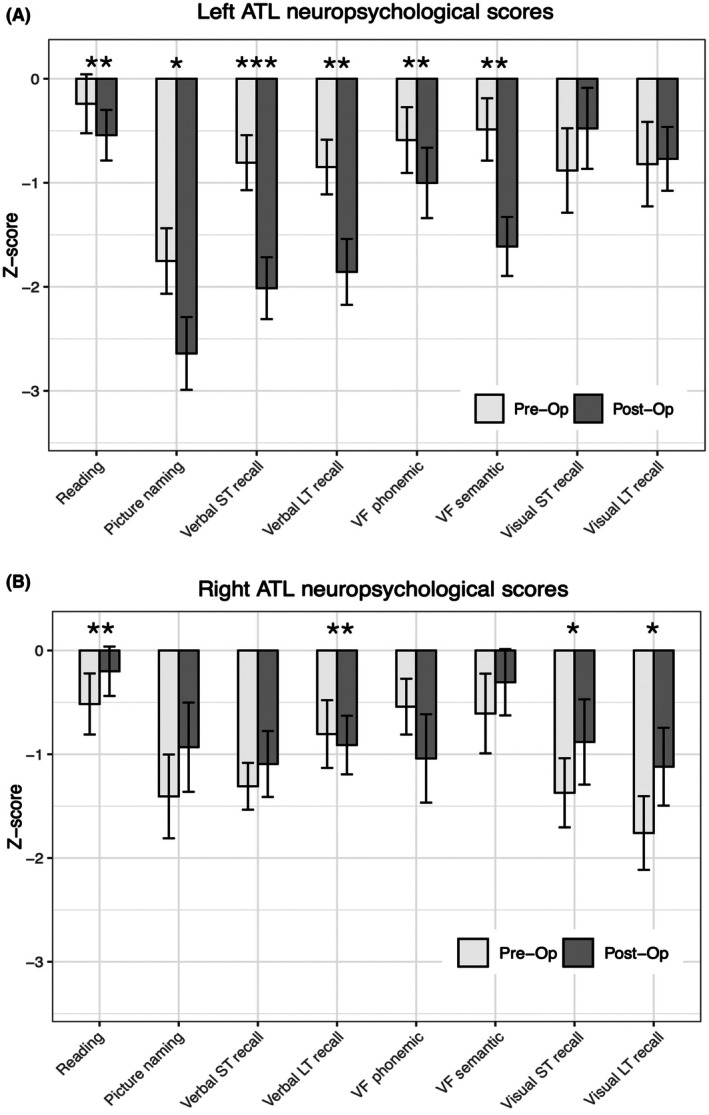
Groupwise neuropsychological status before and after surgery. All values are *Z*‐scores relative to age‐ and education‐matched controls. *p < .05 and false discovery rate‐corrected *q* < .10. **q < .05. ***q < .01. ATL, anterior temporal lobectomy; LT, long‐term; ST, short‐term; VF, verbal fluency.

As a group, left ATL patients demonstrated statistically significant lower postoperative than preoperative scores in picture naming (*t* = 2.73, *p* = .014, FDR‐adjusted *q* = .047), semantic fluency (*t* = 4.63, *p* = .0027, *q* = .0044), verbal learning (*t* = 3.90, *p* = .0014, *q* = .011), verbal recall (*t* = 2.85, *p* = .011, *q* = .046), and word reading (*t* = 2.71, *p* = .018, *q* = .047). Interestingly, right ATL patients demonstrated higher postoperative than preoperative scores in picture naming (*t* = −2.99, *p* = .010, *q* = .046) and word reading (*t* = −2.65, *p* = .021, *q* = .048). All other tests did not show significant change (see Figure 3).

### 3.4 Neuropsychological change and hippocampal resection

**FIGURE 4:**
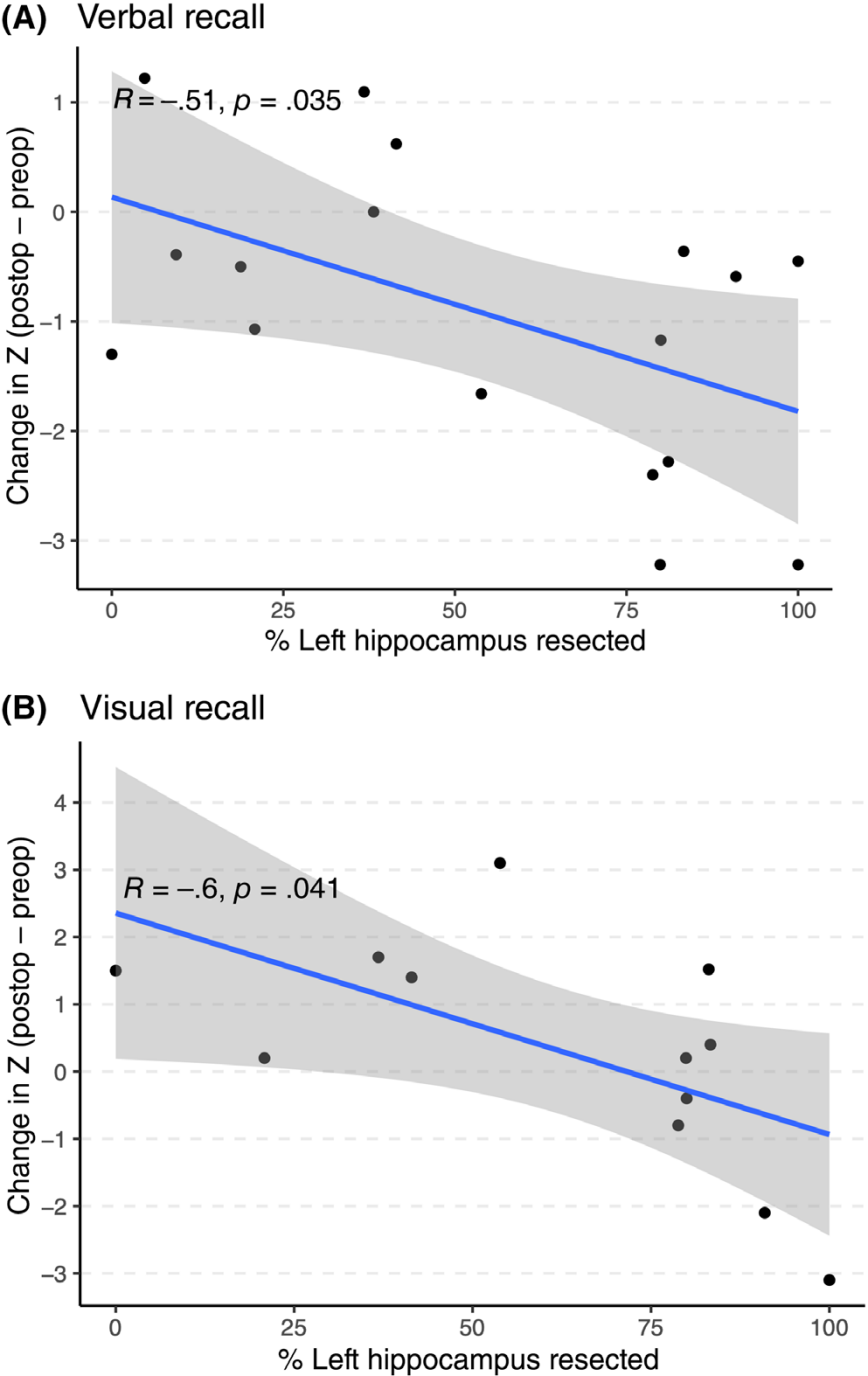
Association between hippocampus resection percentage with change in (A) verbal and (B) visual delayed recall following left anterior temporal lobectomy. As the percentage of hippocampus to be resected increases, verbal and visual memory outcomes decrease. Note that the results for verbal recall demonstrate no change when the left hippocampus is spared, and an increasing deficit (change < 0) as more hippocampus is resected. In contrast, for visual recall, an improvement (change > 0) is demonstrated when left hippocampus is spared, with this improvement fading as more hippocampus is resected, and eventually becoming a deficit.

For left ATL, greater extent of hippocampal resection was associated with a larger reduction in verbal recall (*ß* = −.019, 95% CI = −.038 to −.0016, adjusted *R* ^2^ = .21, *p* = .035) and greater reduction in visual delayed recall (*ß* = −.033, 95% CI = −.064 to −.0016, adjusted *R* ^2^ = .29, *p* = .041; Figure 4). A similar relationship was observed, yet did not reach significance, for visual short‐delay recall (*ß* = −.011, 95% CI = −.062 to .0028, adjusted *R* ^2^ = .11, *p* = .067; Figure S1). No other relationships approached significance for left ATL (*p* > .15 for all). For right ATL, percent of hippocampal resection was not significantly associated with neuropsychological change in any domain, nor were any near‐significant trends observed (*p* > .15 for all). A complete view of all relationships between extent of hippocampal resection and neuropsychological variables can be found in Figures S1 and S2.

### 3.5 Seizure outcomes

Of 34 patients included in this study, 24 (70.6%) were seizure‐free (Engel I) at last follow‐up. Among the subset of patients with at least 2 years of postoperative follow‐up (*n* = 27), 18 (66.6%) remained seizure‐free, consistent with established benchmarks for surgical outcomes in temporal lobe epilepsy. Crucially, there was no significant difference in extent of hippocampal resection between seizure‐free and non‐seizure‐free patients (see Tables 2, 3, 4). The rate of seizure freedom did not significantly differ between patients who underwent left ATL and those who underwent right ATL (see Table 1). Furthermore, a comparison of clinical variables revealed no significant differences between seizure‐free and non‐seizure‐free patients with respect to the percentage of hippocampal resection, age at the time of surgery, age at seizure onset, or duration of epilepsy (see Tables 2, 3, 4). These findings suggest that seizure outcomes in this cohort were not strongly influenced by these demographic or surgical factors.

**TABLE 2:** Seizure‐free and non‐seizure‐free patients did not differ in their age at surgery, age at seizure onset, preoperative HV, years of seizures, or percent of resection.

	Engel I, *n* = 24		Engel II–IV, *n* = 10		*T*	*p*
	Mean	SD	Mean	SD		
Age at surgery, years	37.3	11.9	34.6	14.2	−.48568	.63
Age at seizure onset, years	22.0	16.0	25.3	16.3	.50416	.62
Preoperative HV	2.41	.73	2.43	.45	.10	.92
Years of seizures	15.3	10.5	9.3	10.0	−1.4517	.17
Percent resection	63.5	33.7	69.7	31.9	.46643	.65

Abbreviation: HV, hippocampal volume.

**TABLE 3:** Seizure‐free and non‐seizure‐free patients did not differ in their neuropsychological change.^a^

	Mean change	SD	Mean change	SD	*T*	*p*
Reading	.45	.47	−.25	.76	−.71	.49
Verbal short recall	−.44	1.27	−.80	1.67	−.54	.60
Verbal long recall	−.71	.99	−.44	1.73	.41	.69
Picture naming	.10	1.25	−.99	.84	−2.48	.024*
Phonemic fluency	−.15	.94	−.39	.67	−.74	.47
Semantic fluency	−.075	1.21	−.91	1.98	−1.10	.30
Visuospatial short recall	.46	.93	1.25	2.19	.69	.53
Visuospatial long recall	.22	1.15	.69	2.24	.48	.65

^a^ The sole exception is picture naming, which declined more in patients who were not seizure‐free than patients who were seizure‐free, although this relationship does not retain significance after false discovery rate correction.

* *p* < .05.

**TABLE 4:** Seizure‐free and non‐seizure‐free patients did not differ in the incidence of hippocampal sclerosis.

	*n*	%	*n*	%	*χ* ^2^ (*df* = 1)	*p*
Hippocampal sclerosis present	8	25%	1	10%	.958	.328

## 4 DISCUSSION

Our results demonstrate that the extent of left hippocampal resection is related to degree of postoperative verbal and visual memory function; patients with higher extent of resection have worse memory outcomes. Importantly, despite tailored resections aiming to preserve hippocampal tissue when feasible, our cohort achieved an overall seizure freedom rate of 70.6% at last follow‐up, and 66.6% for patients with 2 or more years of follow‐up. This aligns with published seizure outcomes for ATL, which range between 62% and 75%.^1^, ^2^, ^3^ These results highlight that individualized resections, guided by SEEG and refined with intraoperative electrophysiology, can achieve seizure control comparable to standard resections while offering the possibility of mitigating cognitive risks. Hippocampal preservation can be achieved without compromising seizure control in appropriately selected patients.

Although previous investigations have explored cognitive outcomes after standard ATL or SAH, none has combined volumetric resection analyses with individualized invasive electrophysiological mapping to precisely tailor surgical extent. This work fills a critical gap in the literature and offers evidence that hippocampal resection volume is a continuous and clinically meaningful predictor of postoperative cognitive outcomes, a clinically useful finding when counseling patients about expected cognitive changes after surgery.

Our findings on visual memory outcomes in left ATL highlight the utility and significance of this approach. Group‐based comparisons (Figure 3) indicated no statistically significant change in visual delayed recall for left ATL patients. Nonetheless, further investigation revealed that the more of the left hippocampus is resected, the more postoperative visual delayed recall is disrupted (Figure 4B). This relationship was not detectable via group‐based comparison, because patients with left ATL and small hippocampal resections experienced some improvements in visual memory, whereas patients with left ATL and large hippocampal resections experienced some decline. Therefore, as a group, their average change was near 0. Yet when examined from a correlation perspective, there is a relationship between left hippocampal resection and visual memory function. These findings are in agreement with prior reports indicating that visuospatial memory functions of the hippocampal formation may not be as strongly right‐lateralized as verbal memory is left‐lateralized.^28^, ^29^, ^30^, ^31^ We emphasize the need to consider potential visuospatial memory impacts, even in dominant hemisphere surgeries where verbal memory risks often predominate preoperative counseling.

### 4.1 Statistical versus clinical significance of changes in neuropsychological performance

We did not collect measures of whether neurocognitive deficits were clinically salient to patients, their families, or their clinical providers. Consider as an example our results regarding changes in word reading performance in left ATL patients. At a group level, there was a statistically significant decrease from preop to postop (*t* = 1.89, *p* = .047), with the magnitude of the effect ~.38 SD. Although the effect is statistically meaningful, a cognitive change of this magnitude may not be clinically significant in all individuals: for a patient who is a trial lawyer, a slight reading impairment may be highly impactful, whereas for another individual, such a change might not affect their quality of life or even be noticed.

Thresholds for defining clinically significant neuropsychological decline differ.^32^, ^33^ One conservative rubric is to assume that a change that is >1 SD is a clinically significant improvement or decline; following that guideline, three of our group‐level results (Figure 1) would be considered clinically significant: reduced semantic fluency and reduced verbal learning after left ATL, and improved visual memory after right ATL. A similar framework suggests that both core findings relating extent of hippocampus resection is related to neuropsychological outcomes, are “clinically meaningful.” For verbal memory, a *ß* of −.019 would mean that resection of 53% or more of the left hippocampal tissue corresponds to a >1‐SD difference in outcome (see Figure 3A). For visual delayed recall, a *ß* of −.033 would mean that resection of 31% or more of left hippocampal tissue corresponds to a 1‐SD difference in outcome (see Figure 3B). These estimates serve as important benchmarks for future prospective research to test the degree to which extent of hippocampal resection has clinically meaningful consequences for cognitive function.

### 4.2 Role of SEEG and intraoperative ECoG

The use of SEEG in our cohort allowed precise three‐dimensional mapping of seizure onset zones and propagation pathways, enabling tailored resections that targeted only the epileptogenic tissue while sparing uninvolved hippocampal segments. This is complemented by intraoperative ECoG, which provided real‐time electrophysiological confirmation of residual epileptogenic activity during resection, further refining surgical boundaries. Together, these tools represent a paradigm shift from purely anatomical resections toward AEC, patient‐specific surgical strategies. Prior studies have emphasized the utility of SEEG in localizing deep mesial temporal onset zones.^12^, ^13^ Our results build upon those prior findings by demonstrating strong cognitive and seizure outcomes when SEEG and ECoG are integrated into surgical decision‐making.

### 4.3 Seizure outcomes in tailored resections

Our cohort achieved an overall seizure freedom rate of 70.6%, with 66.6% seizure freedom in those with ≥2 years of follow‐up. These results align with published outcomes for ATL, which typically report seizure freedom rates ranging from 62% to 75%.^1^, ^2^, ^3^ The comparable seizure outcomes in our study underscore the effectiveness of SEEG‐guided and intraoperative ECoG‐tailored resections for achieving seizure control, despite efforts to preserve hippocampal tissue when feasible. Tailored approaches are capable of successfully differentiating more severe cases with more extensive EZs from cases with EZs that do not extend as far into the hippocampus.

### 4.4 Limitations

A principal limitation of the current study is the sample size. Future studies with larger prospectively enrolled cohorts can test interactions with factors that may influence the relationship between hippocampal integrity and cognitive outcome.^6^, ^34^ These variables include seizure freedom, preoperative cognitive status, presence or absence of hippocampal sclerosis, and hemispheric dominance. We qualitatively report on these factors below, but our study is not adequately powered to test them as possible moderating factors.

One future consideration is the potential interaction of resection extent and seizure freedom to influence postoperative neuropsychological performance. A recent long‐term study of neuropsychological outcomes following pediatric epilepsy surgery demonstrated that seizure freedom has benefits for cognitive functioning, an important consideration when seeking to maximize seizure freedom and minimize functional impact.^35^

Another important clinical variable is the presence or absence of hippocampal sclerosis on MRI. Hippocampal sclerosis has long been established as a relevant predictor of cognitive changes following ATL, particularly left ATL.^36^ For patients with hippocampal sclerosis, a large percentage of the hippocampus may represent a comparatively small absolute volume of tissue. Although our study is not adequately powered to consider the interaction of hippocampal sclerosis with extent of resection, we have included information on the hippocampal sclerosis status (based on MRI) of each patient in Table S1. Figures S3 and S4 depict the relationship between absolute volume of hippocampal resection and cognitive change, which are qualitatively similar to the percentage‐based results we report; as noted, however, the *N* is not sufficient to test interactions between absolute and relative volume of resection in determining outcomes.

Hemispheric dominance is also likely to play a role in cognitive outcomes. For instance, preoperative fMRI has been shown to contribute to explaining variance in naming outcome after left ATL surgery.^37^ Although all of our patients were deemed to have language and verbal memory presence in the left hemisphere, some were also indicated to have verbal memory presence in the right hemisphere; this current sample was not adequately powered to address any potential interaction of those two variables. Investigation of these relationships in patients with right‐dominant language will be crucial to future prospective trials.

Finally, there are longitudinal considerations that apply to all aspects of this design: hippocampal volume measurements, neuropsychological measurements, and seizure freedom outcomes. Our study calculated hippocampal volume change from before surgery to <48 h after surgery, too soon to be indicative of chronic postoperative hippocampal atrophy. Even a completely nonresected hippocampus is likely to experience tissue atrophy in the months following surgery, and this atrophy, measured at 6 months, has been found to correlate with postoperative memory decline.^38^ Second, in terms of postoperative neuropsychological outcome, our evaluations occurred at a mean of 7 months, typically occurring between 5 months and 1 year, which may not capture all postoperative cognitive recovery or decline. Third, extended follow‐up of seizure freedom will be beneficial to future work. Although the majority of seizure recurrences occur in the first 2 years, they can also occur much later on.^39^

## 5 CONCLUSIONS

We investigated the relation between extent of hippocampal tissue resection and changes in neuropsychological performance in tailored, AEC‐guided anterior temporal lobe resections. We found that smaller extent of hippocampal resection is associated with better cognitive outcomes in both visual and verbal delayed recall, with seizure outcomes commensurate with the current reported standard. These results highlight the clinical utility of tailored hippocampal resections, guided by detailed AEC correlations and appropriate noninvasive/invasive monitoring, particularly in the left hemisphere. The hippocampus is not a functionally unitary structure, and hippocampal resection should not be treated as an “all‐or‐nothing” phenomenon.

## AUTHOR CONTRIBUTIONS

**Eliza M. Reedy:** Conceptualization; data curation; formal analysis; investigation; writing—original draft preparation; writing—review & editing. **Emma Robinson:** Data curation. **Thandar Aung:** Conceptualization; writing—review & editing. **Catherine Liégeois‐Chauvel:** Conceptualization; writing—review & editing. **Danielle R. Carns:** Investigation. **Natalie Sherry:** Investigation. **Luke C. Henry:** Conceptualization; investigation; writing—review & editing. **Bradford Z. Mahon:** Conceptualization; funding acquisition; writing—review & editing. **Arka N. Mallela:** Conceptualization; investigation; funding acquisition; supervision; writing—review & editing. **Jorge A. Gonzalez‐Martinez:** Conceptualization; funding acquisition; supervision; resources; writing—review & editing.

## FUNDING INFORMATION

This work was supported in part by the Clinical and Translational Sciences Institute at the University of Pittsburgh (grant number UL1‐TR‐001857), NIH F32DC020644 to A.N.M., NIH R01NS122927 to J.A.G.‐M., NIH R01NS089069 to B.Z.M., and funding via the Physician‐Scientist Institutional Award from the Burroughs Wellcome Fund and NIH UE5NS134521 to the University of Pittsburgh. Funding sources were not involved in the data collection, analysis, writing of the manuscript, or decision to submit it for publication.

## CONFLICT OF INTEREST STATEMENT

E.M.R. and J.A.G.‐M. have served as paid consultants for DIXI Medical US. B.Z.M. is an inventor of intellectual property PCT/US2019/064015, which describes a process for developing predictive analytics in neurosurgery, and is a cofounder and CSO of MindTrace Technologies, which licenses said intellectual property from Carnegie Mellon University. The remaining authors have no conflicts of interest. We confirm that we have read the Journal's position on issues involved in ethical publication and affirm that this report is consistent with those guidelines.

## Supporting information


**Data S1.** Supplementary figures.

**Data S2.** Supplementary tables.

## Data Availability

The data that support the findings of this study are openly available in Figshare at 10.6084/m9.figshare.29959622.v1.
