# Supplementary material for: Graded extent of hippocampal resection is related to neuropsychological outcomes in temporal lobe epilepsy surgery
Source: Epilepsia. 2026 Feb 26;67(6):2755–67. doi: 10.1002/epi.70162 (PMC13285241; doi:10.1002/epi.70162)

Supplementary Figure 1. Representative examples of partial or complete hippocampal sparing, with segmentation masks.

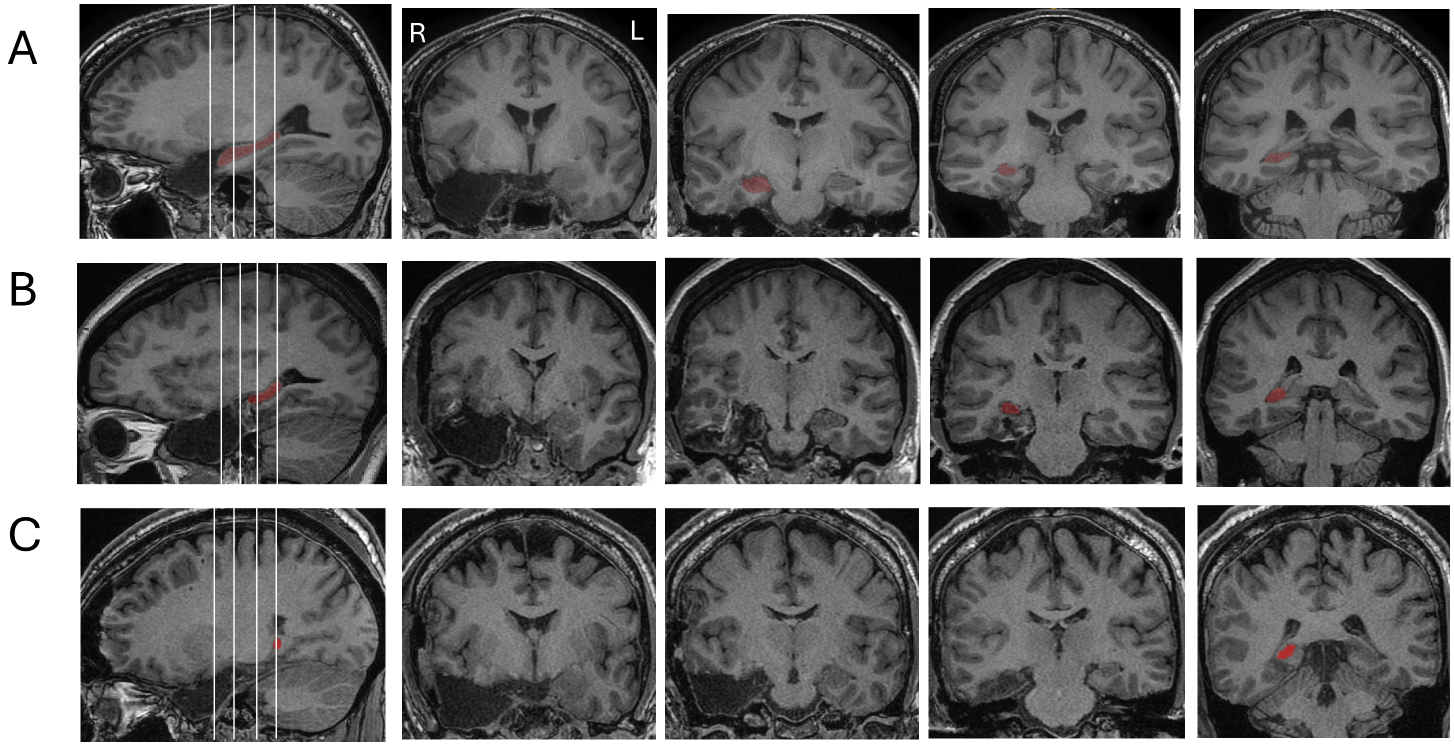

Supplementary Figure 2. Scatterplots of all neuropsychological outcome variables for left-sided ATL

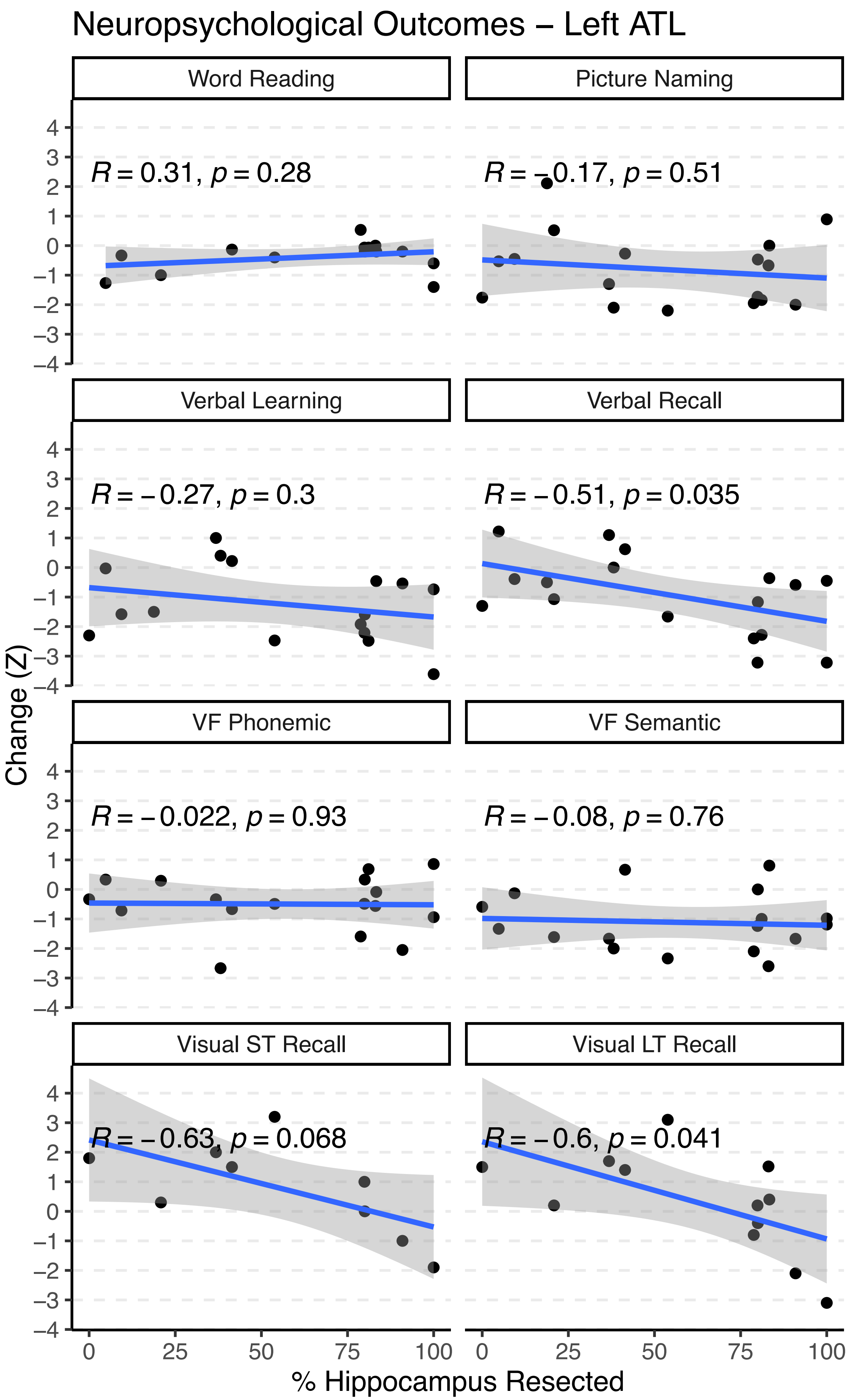

Supplementary Figure 3. Scatterplots of all neuropsychological outcome variables for right-sided ATL

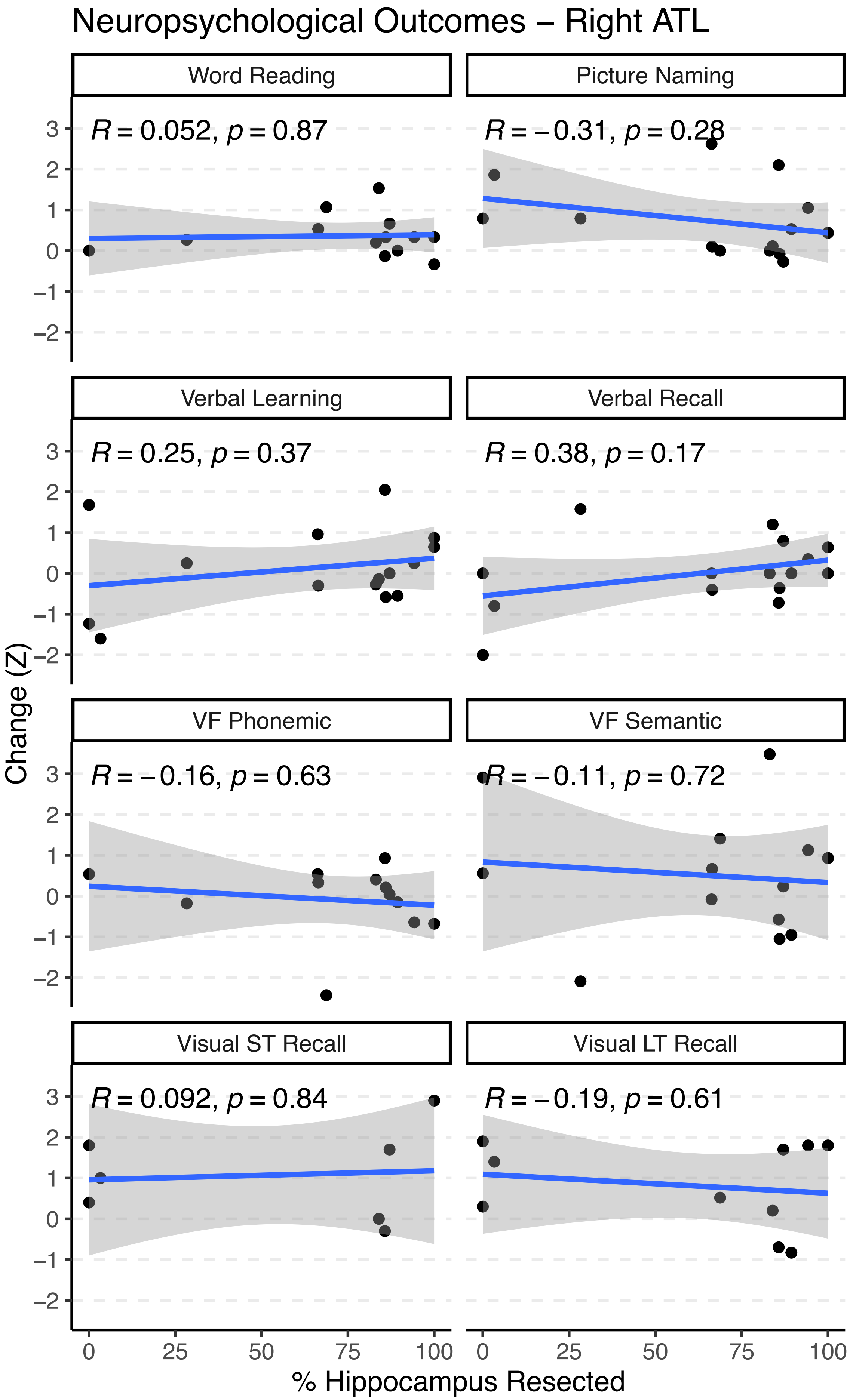

Supplementary Figure 4. Scatterplots of all neuropsychological outcome variables for right-sided ATL, by absolute tissue volume resected.

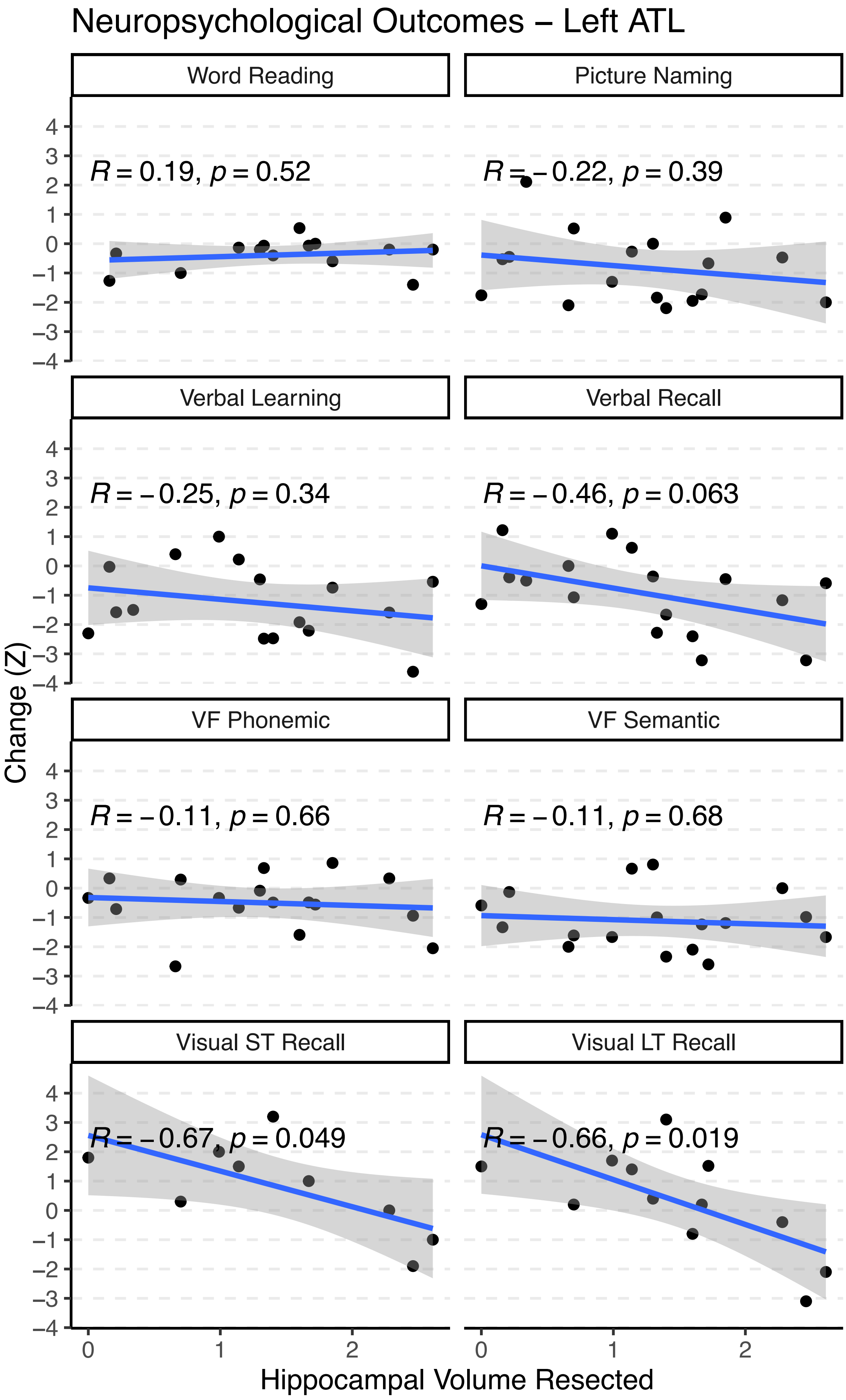

Supplementary Figure 5. Scatterplots of all neuropsychological outcome variables for right-sided ATL, by absolute tissue volume resected.

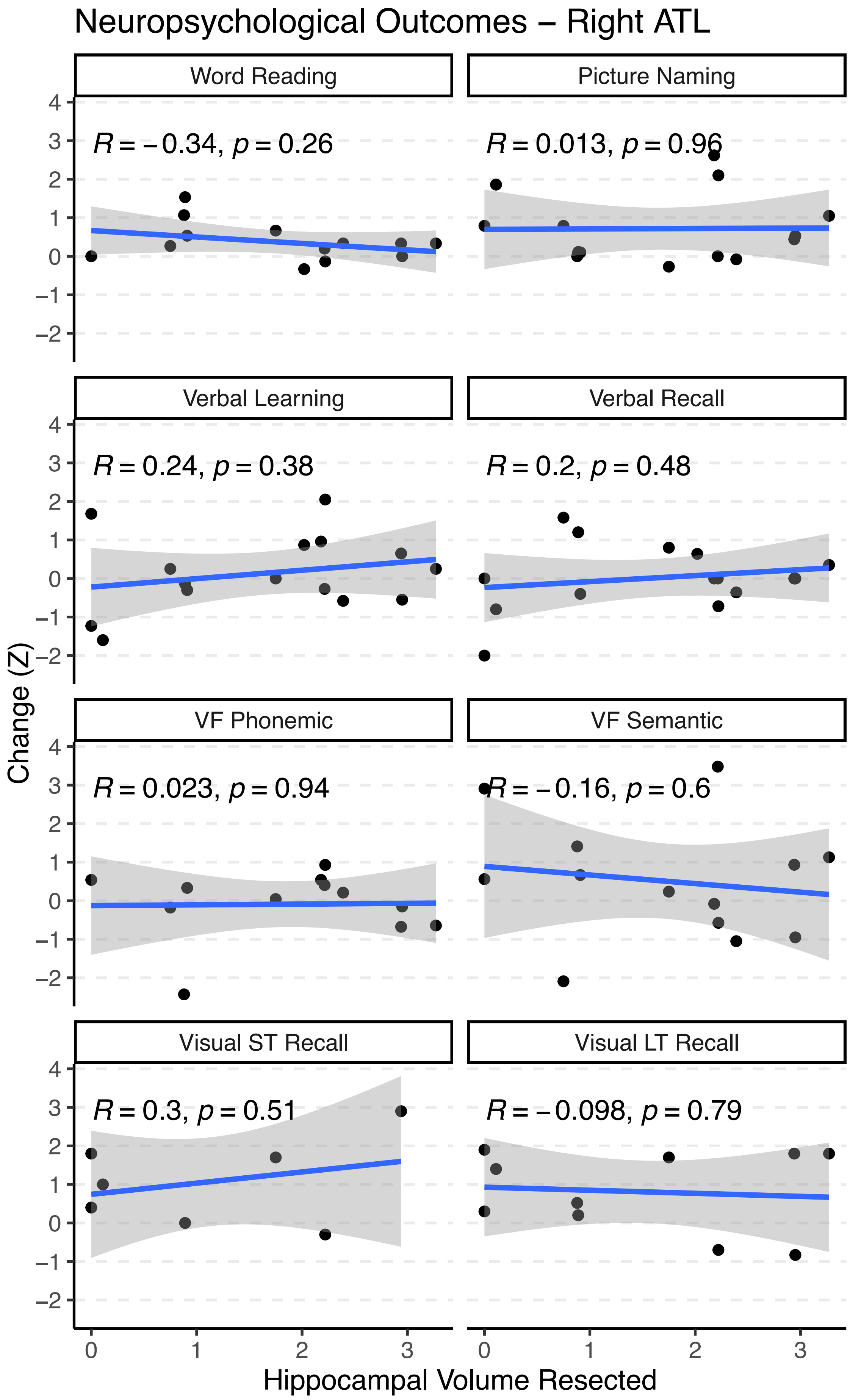

Supplement: Supplementary file 1 — Data S1. Supplementary figures. [file EPI-67-2755-s001.pdf]
