# Supplementary material for: Graded extent of hippocampal resection is related to neuropsychological outcomes in temporal lobe epilepsy surgery
Source: Epilepsia. 2026 Feb 26;67(6):2755–67. doi: 10.1002/epi.70162 (PMC13285241; doi:10.1002/epi.70162)
Supplement: Supplementary file 2 — Data S2. Supplementary tables. [file EPI-67-2755-s002.pdf]

Supplementary Table 1. Clinical characteristics of all patients. For patients who frequently experience multiple seizure types, the most frequent type is listed first. HS = hippocampal sclerosis. ASMs = anti-seizure medications. MEG = magnetoencephalography.

ID	Age at Surgery	Age at Seizure Onset	Aura/Semiology	Surgical side	Preop Seizure Frequency	ASMs at Pre	ASMs at Post	MEG Verbal Memory	HS	Preop SEEG	Hippocampal SEEG electrodes
**1**	19	1	No aura<br>FAS	L	1/month	2	2	-	N	Y	L head
**2**	26	57	No aura<br>FIAS	R	5/year	2	2	B	Y	Y	R head, R body
**3**	64	37	Aura: Left facial numbness<br>FIAS	L	1-2/month	2	2	L	N	Y	L head, R head
**4**	45	40	Aura: Confusion, L arm numbness<br>FBTC	L	1-2/month	2	3	L	Y	Y	L head
**5**	24	23	No aura<br>FIAS	R	1/month	4	4	L	N	Y	R head
**6**	23	34	Aura: sweaty, anxious<br>FBTC	R	3-4/month	2	2	L	N	Y	R head, R body
**7**	45	11	No aura<br>FAS	L	1/day	3	3	B	N	Y	L head, L body
**8**	31	65	Aura: nausea, chills, metallic taste<br>FAS	R	1/month	2	2	-	N	Y	R head
**9**	43	27	Aura: “zoning out”<br>FIAS	L	3-4/month	2	2	L	N	Y	L head, L body
**10**	52	19	Aura: fear<br>FAS or FIAS	L	2-3/month	2	2	-	N	Y	L head, L body
**11**	42	25	Aura: Epigastric rising<br>FBTC	L	6-8/year	3	3	B	N	Y	L head, L body
**12**	25	13	Aura: Nausea<br>FIAS or FBTC	R	8-9/year	2	2	L	Y	Y	L head, L body
**13**	39	26	Aura: Déjà vu<br>FIAS or FAS	R	6-7/month	2	1	B	N	Y	R head, R body
**14**	28	34	Aura: Déjà vu<br>FBTC or FIAS	R	1-2/month	3	3	-	N	Y	R head, R body
**15**	68	4	Aura: Fear, Déjà vu, aphasia<br>FIAS	L	4-5/day	4	3	L	N	Y	L head
**16**	29	60	No aura (out of sleep)<br>FBTC	L	6/year	2	2	L	Y	Y	L head, L body
**17**	31	20	Aura: tingling in stomach<br>FIAS	R	0-4/day	2	2	L	Y	Y	R head, R body

**18**	20	28	Aura: fear, tachycardia<br>FIAS or FBTC	L	1/1-2 months	2	3	-	N	Y	L head, L body
**19**	49	23	Aura: feeling "weird", things don't sound right<br>FAS	L	2-4/month	4	3	-	Y	Y	L head
**20**	37	15	Aura: blurry vision, numbness in mouth<br>FBTC	R	1-2/week	3	3	-	N	Y	R head, R body
**21**	35	21	Aura: occasional blurry vision<br>FBTC or FIAS	L	1-2/month	2	2	-	N	Y	L body
**22**	30	27	Aura: fear, lightheadedness, ringing in ears<br>FAS or FBTC	L	1/day	3	3	-	N	Y	L head, L body
**23**	38	8	No aura<br>FIAS or FBTC	R	1/week	1	1	L	N	N	-
**24**	37	18	Aura: Confusion, mumbling, right hand movements<br>FBTC	R	4-6/year	3	3	-	Y	N	-
**25**	64	21	No aura<br>FIAS	R	1/month	2	1	L	N	N	-
**26**	38	10	Aura: vision changes<br>FIAS or FBTC	L	4-6/month	2	2	-	N	Y	L head, L body
**27**	25	33	Aura: epigastric rising, nausea, déjà vu, blurry vision, dizziness, confusion<br>FAS	L	1/day	1	1	L	Y	N	-
**28**	24	35	Aura: tachycardia, distorted perception of surroundings<br>FAS	R	3-4/year	2	2	-	Y	Y	R head, R body
**29**	45	0	No aura<br>FIAS or FBTC	R	4-5/month	5	4	L	N	N	-
**30**	23	60	Aura: "buzzing" in chest<br>FIAS	R	2-3/year	2	2	-	N	Y	R head, R body
**31**	28	2	Aura: dizziness, heat and pressure in heat<br>FIAS	R	3/week	3	3	-	N	Y	R body
**32**	62	16	Aura: Déjà vu<br>FIAS	L	2-4/month	1	1	L	N	Y	L head, L body

**33**	27	18	Aura: racing thoughts, tachycardia, fear<br>FAS	L	2-7/week	3	3	L	N	Y	L head, L body
**34**	38	5	No aura<br>FIAS	L	3/month	3	3	-	N	Y	R head, R body
